# Editorial: Molecular and immune influences in the progression of gliomas

**DOI:** 10.3389/fonc.2023.1102445

**Published:** 2023-02-01

**Authors:** Karan Malik, Noreen Mian, Maria Caffo, Haotian Zhao

**Affiliations:** ^1^ Department of Biomedical Sciences, College of Osteopathic Medicine, New York Institute of Technology, Old Westbury, NY, United States; ^2^ Department of Biomedical and Dental Sciences and Morpho-Functional Imaging, Unit of Neurosurgery, Messina, Italy

**Keywords:** glioma, tumor microenvironment, biomarker, immune suppression, epithelial to mesenchymal transition, autophagy, long non-coding RNA, microRNA

Diffuse gliomas are a heterogeneous group of tumors that represent the most prevalent and lethal primary tumors of the brain (1). High-grade gliomas are among the most difficult cancers to treat, for which first-line therapy – a combination of maximal surgical resection, radiotherapy, and chemotherapy with temozolomide (TMZ) – remains unchanged over a decade with few effective targeted therapies (2). Histologically, gliomas are categorized into four grades by WHO. Grade I gliomas usually grow slowly and behave in a more benign manner. Grade II and III gliomas can grow more rapidly and frequently require more aggressive treatment. Grade IV gliomas, also known as glioblastoma (GBM), is the most common and clinically aggressive gliomas, with median overall survival for patients with GBM at ~ 15 months (2). At the genomics level, gliomas in adults comprise two major groups based on the mutational status of isocitrate dehydrogenase genes *IDH1* and *IDH2*. Although IDH-mutant gliomas usually start as lower histologic grade tumors with improved prognosis, they often progress to higher grades. In contrast, IDH-wild-type gliomas typically present as GBM (2).

Multi-omics and recent technological advances such as single-cell techniques have provided a detailed and expanding appreciation of glioma intertumoral and intratumoral heterogeneity at genetic and epigenetic levels (3). The interactions with these heterogeneous components result in profoundly diverse phenotypic outcomes that contribute to adaptative responses and therapeutic resistance in this group of highly resilient disease. Importantly, heterogeneous cell populations within the tumor microenvironment (TME) represent an important aspect of glioma pathogenesis. Besides neuroglial cell types, gliomas are enriched for tumor-associated macrophages (TAM), while the maturation of natural killer (NK) cells is also affected in different glioma subtypes. The abundance of TAMs and low levels of infiltrating T cells constitute an immunosuppressive TME for adult gliomas (**Figure 1A**) (3, 4). With recent success of immunomodulatory therapy in diverse cancer types, there is significant interest in the study of immune regulation in the TME of glioma, and its implication in immunotherapy.

**Figure 1:**
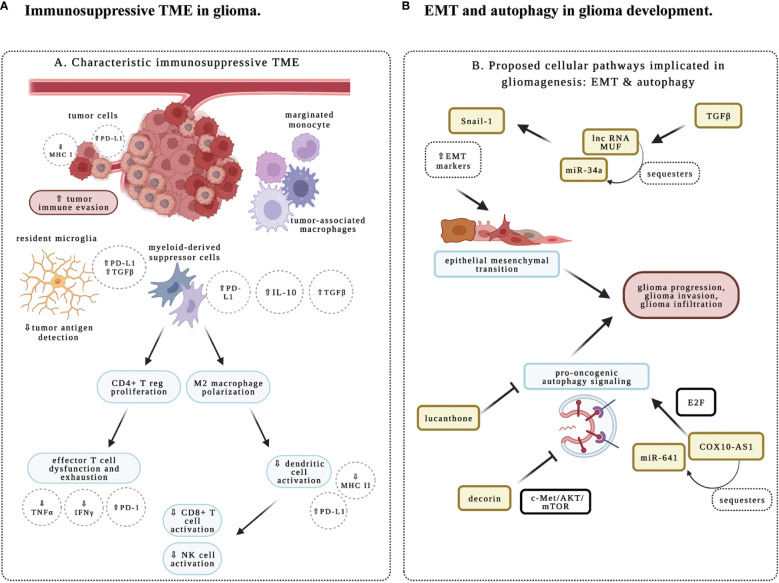
Schematic representation of immune and molecular regulation in glioma microenvironment. **(A)** Immunosuppression in glioma tumor microenvironment (TME). Immune responses are impacted by diverse cell types in the TME. Studies in the research topic highlight the importance of characterizing the immune phenotype in glioma. Tumor-associated macrophages (TAMs) include resident microglia, monocytes that will differentiate into M2- versus M1-predominant macrophages, and myeloid-derived suppressor cells (MDSCs), which are peripherally derived immunocytes that marginate through the blood-brain barrier. These cells are characteristic of an immunosuppressive TME and secrete key growth factors including interleukin 10 (IL-10) and transforming growth factor-beta (TGFß) to support an oncogenic niche. Cells of lymphoid origin, including T cells and natural killer (NK) cells; as well as other peripheral infiltrating cells, such as dendritic cells (DCs), are reported to be sparse within TME. MDSCs have been shown to prime CD4+ regulatory T cells (Treg) for an immunosuppressive and anergic role within gliomas. MDSC-primed Treg suppresses CD8+ effector T cell response, and contributes to effector T cell exhaustion through the downregulation of major histocompatibility complex class I molecules (MHC-I), suppression of signaling by interferon (IFN)-γ and tumor necrosis factor (TNF)-α, and upregulation of programmed cell death ligand 1 (PD-L1). The M2-polarized macrophages typically downregulate the expression of MHC-II and PD-L1 to suppress the activation of CD8+ T cells, NK cells, and DCs in the TME, thereby contributing to the immunosuppressive properties of TME. **(B)** Epithelial to mesenchymal transition (EMT) and autophagy in glioma. Studies in this research topic illustrate how cellular and molecular aspects of autophagy and EMT facilitate glioma infiltration, recurrence, and progression. COX10-AS1 is a lnc-RNA that is shown to upregulate pro-oncogenic autophagy signaling mechanisms *via* the E2F family of proteins. Decorin is an extracellular matrix component that inhibits autophagy *via* c-Met/Akt/mTOR signaling, and may function as a novel therapeutic target. Lucanthone is effective in inhibiting autophagy, glioma cell proliferation and survival. And TGFß within the TME is shown to promote EMT, as evident by upregulation of EMT markers like Snail-1 *via* miR-34a sequestration by the lnc-RNA MUF. Created with BioRender.com.

This topic introduces current developments in molecular and immune-mediated mechanisms in gliomas. One important goal was to investigate potential biomarkers in gliomas which may be useful tools in predicting behaviors of subpopulations of immune infiltrates and in determining which glioma subtypes may be amenable to genotoxic and immunomodulatory therapies. A systematic review was performed investigating proteins, nucleic acids, circulating cells, and metabolites, as potential blood-based biomarkers for glioma (Ali et al.). Around 200 targets are categorized according to their clinical utility in predicting recurrence, identifying highly infiltrative glioma subtypes, and optimizing therapeutic management. The study found that panels of microRNAs and proteins are the most promising biomarkers, while a selection of single biomarkers may also be useful. Consistently, several manuscripts drew on multiple modalities including the study of institution-specific patient cohorts, bioinformatics, and multi-omics data from The Cancer Genome Atlas (TCGA) and Chinese Glioma Genome Atlas (CGGA). TAMs in glioma can arise from resident microglia or monocytes of peripheral circulation (4). They are engaged in immunosuppression and have unique gene signatures for macrophage activation and increased chemokine/cytokine signaling, which is associated with poor prognosis in glioma patients (**Figure 1A**) (3, 4). CHI3L2 (Chitinase-3-Like Protein 2) is a member of chitinase-like proteins in the glycoside hydrolase 18 family (5), and SIGLEC9 (sialic acid-binding Ig-like lectin-9) is a new member of the Siglec subgroup of the immunoglobulin superfamily expressed in monocytes, neutrophils, T cells and NK cells (6). Two research teams found respectively that elevated levels of CHI3L2 (Liu et al.), or SIGLEC9 (Xu et al.), are associated with poor prognosis and increased immune infiltrates in glioma. CHI3L2 expression is closely related to different activation states of TAMs and induces the apoptosis of CD8+ T cells (Liu et al.). Similarly, SIGLEC9 expression is positively correlated with myeloid-derived suppressor cell infiltration, immune suppression, TAM proliferation and functions (Xu et al.). Therefore, high levels of CHI3L2 or SIGLEC9 could act as unfavorable prognostic factors in glioma patients.

The interactions of malignant cells with immune cells represent critical events in tumor progression (3, 4). However, the molecular details underlying these interactions remain unclear. Three groups discovered that the expression of Tumor necrosis factor receptor superfamily member 12A (TNFRSF12A) (Zhang et al.), methyltransferase like 7B (METTL7B) (Xiong et al.), or Caspase 6 (CASP6) (Guo et al.) in tumor cells is associated with reduced survival in glioma patients, respectively. Further analysis revealed a significant correlation of their expression with immune cell infiltration and immune checkpoints. Knockdown of METTL7B (Xiong et al.) or CASP6 (Guo et al.) inhibits glioma proliferation, suggesting that they contribute to the progression of glioma. These genes could be viable prognostic biomarkers and potential immunotherapeutic targets in glioma.

Predicting the infiltrative behavior of glioma is difficult from a clinical-translational perspective. A prognostic and diagnostic MRI protocol to screen for aggressive and invasive gliomas has been proposed (Li et al.). In a single-center case series, it was shown that T2/FLAIR abnormality could be an indicator of GBM progression, especially for new lesions disseminating from primary sites. It should be noted that these studies report preliminary analysis of candidate indicators of tumor progression and prognosis.

One of the major obstacles to successful treatment of glioma arises from its invasive behavior that enables escape from complete surgical resection and chemo- and radiation therapy (2). Recent work indicates that epithelial to mesenchymal transition (EMT) represents a critical state that promotes glioma infiltration and progression (**Figure 1B**) (7, 8). One group showed that long non-coding RNA (lncRNA) MUF is specifically upregulated by TGFβ (Shree et al.). LncRNA-MUF functions as a sponge for microRNA 34a (miR-34a), promoting the expression of EMT markers. Knockdown of lncRNA-MUF reduces proliferation, migration, and invasion of glioma cells, and sensitizes them to TMZ-induced apoptosis.

Autophagy has two counterbalancing arms in cancer: one that impairs proliferation and invasion, while the other may contribute to tumor progression, invasion, and EMT (**Figure 1B**) (9, 10). Decorin, a proteoglycan in the extracellular matrix, downregulates the expression of EMT markers, suppresses glioma cell migration and invasion (Jia et al.). These effects are achieved through inhibiting autophagy by activating the c-Met/AKT/mTOR axis. In agreement, it has been demonstrated that COX10-AS1, a lncRNA associated with autophagy, promotes glioma progression by sequestering miR-641 to regulate E2F6 (Liu et al.). To complement this work, another study showed that lucanthone, an orally bioavailable and anti-schistosomal agent, acts as an autophagy inhibitor in glioma cells to enhance TMZ efficacy, and suppresses the growth of glioma cells (Radin et al.). Therefore, the unique biology of glioma invasion and progression revealed by these studies will provide potential therapeutic targets.

In summary, the Research Topic of “Molecular and Immune Influences in the Progression of Gliomas“ presents novel preclinical and clinical work in glioma progression and resistance. The diverse work will contribute to developmental therapeutics and the search for clinically adaptable biomarkers to evaluate the anti-glioma therapy, to assess disease response, and to monitor for resistance and recurrence. These studies highlight the importance of multimodal and multidisciplinary approaches in the study of glioma.

## Author contributions

All authors listed have made a substantial, direct, and intellectual contribution to the work, and approved it for publication.
